# Inhibitory Effects of Antimicrobial Peptides on Lipopolysaccharide-Induced Inflammation

**DOI:** 10.1155/2015/167572

**Published:** 2015-11-03

**Authors:** Yue Sun, Dejing Shang

**Affiliations:** ^1^School of Life Science, Liaoning Normal University, Dalian 116081, China; ^2^Liaoning Provincial Key Laboratory of Biotechnology and Drug Discovery, Liaoning Normal University, Dalian 116081, China

## Abstract

Antimicrobial peptides (AMPs) are usually small molecule peptides, which display broad-spectrum antimicrobial activity, high efficiency, and stability. For the multiple-antibiotic-resistant strains, AMPs play a significant role in the development of novel antibiotics because of their broad-spectrum antimicrobial activities and specific antimicrobial mechanism. Besides broad-spectrum antibacterial activity, AMPs also have anti-inflammatory activity. The neutralization of lipopolysaccharides (LPS) plays a key role in anti-inflammatory action of AMPs. On the one hand, AMPs can readily penetrate the cell wall barrier by neutralizing LPS to remove Gram-negative bacteria that can lead to infection. On the contrary, AMPs can also inhibit the production of biological inflammatory cytokines to reduce the inflammatory response through neutralizing circulating LPS. In addition, AMPs also modulate the host immune system by chemotaxis of leukocytes, to promote immune cell proliferation, epithelialization, and angiogenesis and thus play a protective role. This review summarizes some recent researches about anti-inflammatory AMPs, with a focus on the interaction of AMPs and LPS on the past decade.

## 1. Introduction

Inflammation is the part tissue defense against the damage factors, and it is an important component of the innate immune system. Innate immune system is a functional and physical barrier against microorganisms which is naturally stimulated by pathogenic organisms through pattern recognition receptors (PRRs) on host cells [1]. The host cells such as monocytes and macrophages are important for innate immune that can be used as the first line and be recruited to the site of infection to defend against the pathogenic bacteria. Some proinflammation cytokines are the main molecule in macrophage-mediated innate immune responses [2].

LPS plays a crucial role in the pathophysiology of inflammation sepsis and shock [3] caused by Gram-negative bacteria. LPS is a major component of the cell wall of Gram-negative bacteria, which can be released during bacterial cell division or death. Once LPS is released into the blood system, it will cause monocytes and phagocytic cells to produce large amounts of cytokines such as tumor necrosis factor-*α* (TNF-*α*), interleukin-6 (IL-6), and interleukin-8 (IL-8). The overexpression of such cytokines can cause multiple organ damage, such as sepsis. Sepsis is considered to be the most common disease causing mortality in Intensive Care Unit (ICU), and there is no effective, safe drug against it. Antibiotics used in the clinical treatment of inflammation have been very common; however, there are many side effects from the use of it. Antibiotics will speed up the release of bacterial LPS by killing bacteria in order to activate the immune system to secrete cytokines and produce endotoxin shock reaction. For this reason, looking for novel anti-inflammatory drugs that can have both antibacterial and neutralizing LPS is very urgent.

Recent studies have found that AMP not only is a broad-spectrum bactericidal agent but also can directly interact with LPS to inhibit the release of inflammatory cytokines and thus induce an anti-inflammatory effect. AMP may be the best choice for new anti-inflammatory drugs. For this reason, AMP is especially attractive which can be considered as a candidate for inflammation therapeutics, because of their potent activity of antibacterial and high affinity for LPS.

So far, there are more than 1000 natural AMPs that have been discovered and isolated [9, 10]. Although these AMPs are derived from various species and differed in sequence, most of them display a net positive charge and have well-defined secondary structures, like *α*-helical or *β*-strand structures [5–8, 11–14]. Amphipathicity and hydrophobicity of AMPs make them easy to interfere with the bacterial cell membrane stability, then causing leakage of bacteria contents. On the other hand, it is found that many antibacterial peptides can also directly neutralize LPS and inhibit the production of inflammatory cytokines, such as TNF-*α*, IL-6, and IL-8, control immune responses, and reduce inflammatory injury through the different immune regulation. This review is mainly focused on the interaction of the anti-inflammatory AMPs and LPS. It also reviewed how AMPs inhibit LPS-induced inflammation.

## 2. LPS-Induced Inflammation

**Figure 1:**
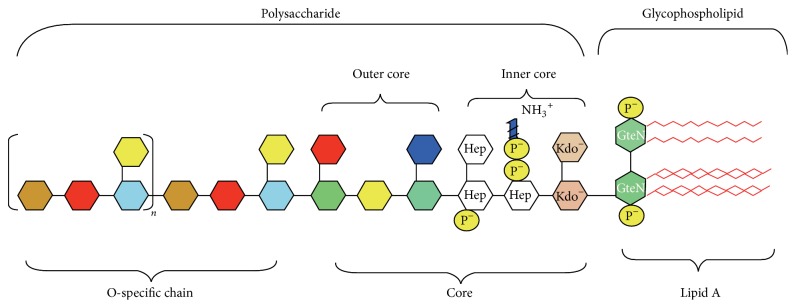
The chemical structure of LPS. Source: [4].

LPS is a major structural and functional component of the Gram-negative bacterial outer membranes. It covers more than 90% of bacterial cell surface. It protects bacteria from antibiotics as a physical barrier. LPS consists of three parts as shown in Figure 1: (i) hydrophobic lipid A that consists of two glucosamines, phosphate, and an amount of fatty acid; (ii) hydrophilic O-antigen which constitutes a polymer of oligosaccharide; and (iii) polysaccharide core which is the connection between the two parts. Lipid A, the conserved portion of LPS, is also the active component of LPS, expressing the endotoxic activity. Lipid A consists of bisphosphorylated glucosamine disaccharide backbone containing six to seven fatty acyl chains per molecule. The core oligosaccharide and the phosphate group of LPS show negative charge, meaning that LPS will exhibit a strong affinity for cation [15].

LPS single molecular weight is about 10 kDa. However, above the critical micellar concentration, LPS can form supramolecular aggregates in aqueous environments, and the molecular weight of this complex can reach 1000 kDa [16].

For the function of the outer membrane (especially LPS), it plays a major role in protecting bacteria from antibiotics. The drug tolerance of bacteria is related to the thickness of LPS layer, which can prevent toxic molecules from entering the bacteria and allow the bacteria to survive in different environments. Meanwhile, the LPS barrier is believed to be stabilized by LPS-associated cations (e.g., Mg^2+^) through salt bridges that neutralize the repulsive forces to link adjacent LPS molecules [17]. It protects bacteria from a variety of host-defense hydrophobic molecules by the oriented and tightly cross-linked leaflet. Second, bacteria adhesion on the surface of the host cell is necessary for the bacteria to infect the host. LPS as an adhesion molecule plays an important role in the pathogenesis of inflammation. Third, LPS can protect bacteria from phagocytes of host cell. Last but not least, LPS is also one of the efficient initiators of innate inflammatory response [18].

**Figure 2:**
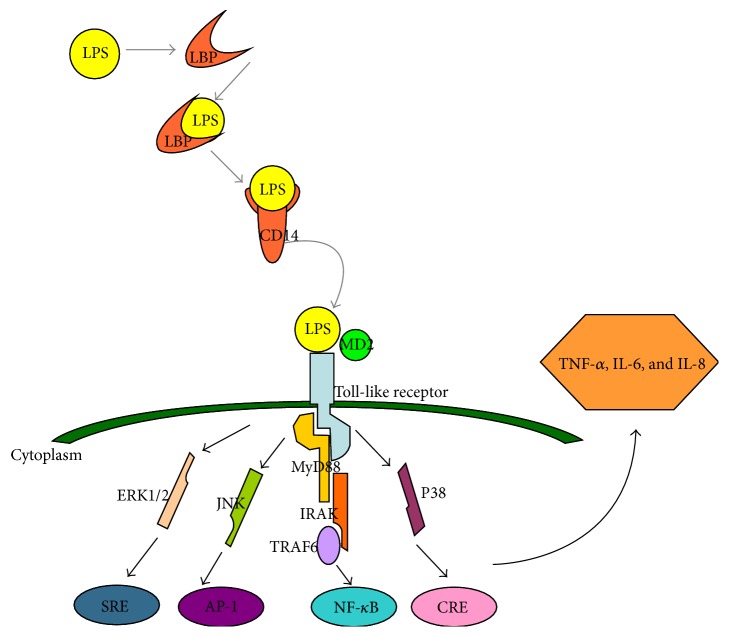
A cartoon outlining the major events in recognition of LPS in the human body.

The important role of LPS in Gram-negative bacteria-induced inflammation has been widely recognized. LPS can interact with several types of host cells and induce inflammation. It is one of the highly conserved pathogen-associated molecular patterns (PAMPs) which is recognized by pattern recognition receptor, inducing the innate immune response. As a result of the antibiotic treatment against bacterial infection, LPS is released from the bacteria during cell death, cell division, or the treatment with antibiotics [19]. Once LPS is released into the blood circulation, it can be recognized by serum protein called LPS-binding protein (LBP) and formed LBP-LPS complexes. LBP is an essential protein that stimulates and amplifies the LPS-induced inflammatory response that is responsible for identifying monocytes. It can recognize LPS and transfers LPS to the cell surface receptor CD14 (mCD14) of mononuclear or phagocytic cell, forming LPS-CD14 complex to activate cells [20, 21]. As CD14 has no transmembrane domain, the LPS-CD14 complex initiates intracellular signaling by interacting with another membrane protein Toll-like receptor 4 (TLR4). TLR4 is a transmembrane protein, which can recognize specific ligands LPS. TLR4 combines with LPS with the help of the MD-2. The TLR pathway activates several different signaling molecules, such as nuclear factor *κ*B (NF-*κ*B) and extracellular signal-regulated kinase (ERK)/c-Jun-NH2-kinase (JNK)/p38 (as shown in Figure 2). The signaling elements induce the secretion of proinflammatory cytokines, including TNF-*α*, IL-1*β*, IL-6, IL-8, NO, and reactive oxygen species (ROS) [22], which can further release the second batch inflammatory cytokines, such as platelet activating factor (PAF), and leukotrienes (LT) [23–25]. However, unbalanced overproduction leads to multiple organ damage and eventually to death.

## 3. The Property of AMP Inhibiting Inflammation

**Figure 3:**
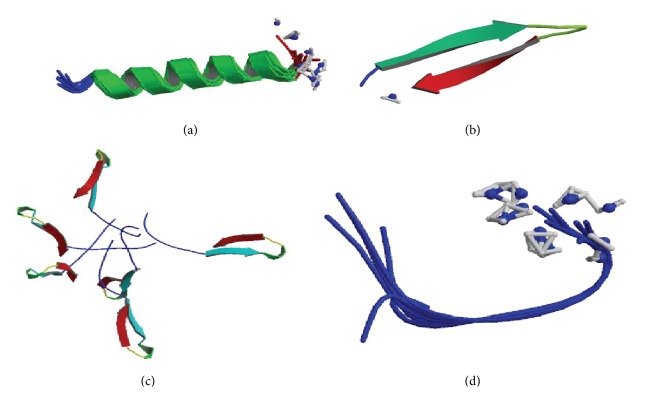
The second structure of antimicrobial peptide. (a) *α*-helical. Source: [5]. (b) *β*-sheet. Source: [6]. (c) Loop. Source: [7]. (d) Extended. Source: [8].

The first antimicrobial peptide, Cecropins, was discovered from the giant silk moth* Hyalophora cecropia* by Swedish scientist G. Boman in early 70s of last century. Until now, more than one thousand of antimicrobial peptides have been characterized in plants and animals, even in bacteria and virus [26–29]. According to their secondary structure, antimicrobial peptides can be divided into four main groups: (i) amphipathic *α*-helices, (ii) *β*-sheet molecules stabilized by two or three disulphide bonds, (iii) extended molecules, and (iv) loop or disordered structures (as shown in Figure 3), with the first two classes being the most common in nature. Despite the different structures and sequences ofantimicrobial peptides, they have some common characteristics: (i) Most of the antimicrobial peptides exhibit amphiphilic structure with hydrophobic surface comprised of nonpolar amino acids and a hydrophilic face containing polar and positively charged residues. (ii) Antimicrobial peptides possess positive charges and have a high content of hydrophobic residues. The structural characteristics of antimicrobial peptides make the interaction with bacterial membranes easy. Cationic antimicrobial peptides could bind to the negatively net charged bacterial cell membranes under the action of electrostatic force and then insert the cell membrane through the amphiphilic and hydrophobic interaction force, by forming an ion channel, eventually causing the cell death [30]. Because of this function, some antimicrobial peptides may protect from infection and inflammation by targeting pathogens directly. Besides, antimicrobial peptides are important components of the innate defense systems of all species, forming the first line of defense with a broad spectrum of biological activity against the pathogenic microorganisms. They can be produced in large amounts at the site of infection or inflammation and quickly eradicate the microorganisms. Alalwani et al. found that, by stimulating with LPS, neutrophils had expressively increased the release of TNF-*α* in cationic AMP CRAMP-deficient animals [31]. Similarly, a deficiency of the sole human cathelicidin LL-37 (consisting of 37 amino acid residues) has increased susceptibility to infections [32, 33]. In addition, the relationship between the expression of AMPs with states of infection and inflammation was found. Lars et al. reported that the expression of many human defense peptides increases during infection and inflammation and decreases the levels of defense that prove the role of antimicrobial peptides in the innate immune system. Some host-defense peptides, which exhibited immune-stimulating activity, have been reported. Neeloffer et al. found that LL-37 can promote the generation of chemokines and inflammatory cytokines IL-1*β* by suppressing small interfering RNA (siRNA) in the presence of GAPDH. GAPDH was identified as a direct binding partner for LL-37 in monocytes. Except for the antibacterial activity and immunoregulation activity, antimicrobial peptides possess anti-inflammatory effect, inhibit the release of proinflammatory cytokines, and alleviate inflammation. Aaron et al. suggested that the human cathelicidin LL-37 inhibits LPS-induced IL-8 from THP-1 monocyte cells. Using enzyme-linked immunosorbent assay (ELISA), B. Fatoumata et al. found that antimicrobial peptide hepcidin inhibits the generation of proinflammation cytokines (such as IL-6, IL-1*β*). Using RT-PCR, Nagaoka et al. showed that human defensin-2 reduced the production of inflammatory cytokines TNF-*α*. However, compared with traditional antibiotics, the capacity of neutralizing LPS of antibacterial peptides made them the candidate of the therapeutic agent against infection or inflammation without side effect. How can AMPs suppress the inflammatory response by interacting with LPS? The interactions between them are divided into three parts.

## 4. The Interaction between AMP and LPS

### 4.1. AMP Binding to LPS

Binding of AMP with LPS plays an important role in both antibacterial activity and anti-inflammatory activity. Li et al. used several approaches including ELISA-based assay, fluorescence correlation spectroscopy (FCS), and surface plasmon resonance (SPR) and found that peptide S3, which was derived from Sushi3 domain of Factor C, could directly bind with LPS. This work demonstrated that antimicrobial peptide S3 dimer has stronger binding capacity to LPS, with 50% LPS-neutralizing capability at a concentration of 1 *μ*M. Magainin 2 binding with LPS by its *α*-helical structure made the leakage of liposomes containing LPS possible, and this effect is weaker in liposome without LPS [34]. rBPI21 is a fragment of neutrophils BPI protein in N-terminal. It is a selective inhibiting Gram-negative bacteria and has a strong affinity for LPS. rBPI21 can cause the leakage of Gram-negative bacteria membrane that is rich in phosphatidylglycerol with the interaction of LPS [35, 36]. How does AMP bind to LPS and what is the key property that influences the binding activity of AMP to LPS?

Hydrophobicity and charge are important factors affecting the bactericidal activity of antimicrobial peptides. These properties determine the interaction between antimicrobial peptides and bacterial phospholipid membrane. As LPS is a content of the phospholipid membrane, hydrophobicity and positive polarity may affect the combination of the antimicrobial peptides and LPS. First, cationic AMPs perform strong electrostatic interactions with the negatively charged LPS in the membrane of Gram-negative bacteria. This enables them to get closer and neutralize the negative charge. Second, the hydrophobicity of AMPs made them easy to embed in LPS micelles. The hydrophobicity and positive charge of antibacterial peptides can increase the ability of binding to LPS. For example, Y. Rosenfeld designed a series of peptides contain twelve amino acids and the fatty acid-conjugated analogues of them consisting of both D- and L-isomers of Leu and Lys at various ratios. He found that adding fatty acid to N-terminus of antimicrobial peptides or using hydrophobic amino acid to replace hydrophilic amino acid can increase their ability to bind with LPS. The different proportions of hydrophobic residues and positively charged residues affect their ability to combine with LPS. The higher the ratio, the stronger the ability to bind with LPS. In addition, removal of hydrophobic residues of antimicrobial peptides significantly weakens their ability to neutralize LPS. Saurabh et al. used Lys to replace Glu in Temporin L showing that hydrophilic amino acid replaced by cationic amino acid can enhance the neutralization of the LPS. Scott et al. reported that antimicrobial peptide CEMA is an analog of CEME (a cecropin-melittin hybrid) with two additional positive charges. He demonstrated that the increased positive charge can strength the ability of CEMA to combine with the LPS [37].

In addition, the distance between the positively charged amino acids is also significant for the binding of LPS. In fact, in LPS-binding peptides, such as Pa4, a member of antibacterial peptide Pardaxins from the mucous glands of sole fishes, the distances between charged amino groups of Lys and Arg range from 12 Å to 15 Å, which is consistent with the interphosphate distance of the lipid A moiety in LPS. There may be a geometrical compatibility between AMPs and LPS conformation [38]. Similarly, Bhunia et al. used NMR in studying the structure and interaction with LPS of AMP MSI-594 (the analog of magainins) and found that the positively charged ammonium (H3N+) groups of Lys residues across the two helices maintain a typical distance range of 12–15 Å [39]. Domadia et al. found that Phe replaced by Ala in MSI-594 made the peptide structure loose, affecting its affinity of LPS [40]. For this reason, the positively charged residues in the peptide can neutralize the negative charge in the lipid A portion of LPS while the hydrophobic residues are inserted into the lipophilic core region with the assistance of the fit structure of AMPs.

### 4.2. AMP Effects on LPS Aggregate Structure

The effects on LPS aggregate structure of AMP are closely related to the capacity of LPS neutralization. Heinbockel and coworkers investigated the effects on LPS aggregate conformations of AMPs, Hb*γ*-35 and Pep19-2.5, by using light scattering technique. It is found that the two peptides interact differently with LPS. In the presence of Hb*γ*-35, LPS aggregates are disaggregated to a cubic form. And Hb*γ*-35 increases the secretion of LPS-induced TNF-*α* in human MNC. Conversely, Pep19-2.5 converted LPS from cubic to a multilamellar form, which brings about the inhibition of TNF-*α* production [41]. Kaconis et al. used a variety of biophysical techniques, like freeze-fracture electron microscopy and synchrotron radiation small-angle X-ray scattering (SAXS), to study LPS neutralization of a series of synthetic peptides. Their work suggests that the capacity of forming LPS multilamellar directly correlates with the inhibition of cytokines production stimulated by LPS [42]. Similarly, by using Cryo-Transmission Electron Microscopy (Cryo-TEM), Chen et al. observed that pure LPS exhibits fibrils with cylindrical forms. However, in the presence of peptide G12.21, which can neutralize LPS efficiently, the LPS structure changes into tightly arranged multilamellar structures [43]. These AMPs can promote LPS forming massive aggregation, which may facilitate the phagocytosis by macrophages, avoiding the activation of cell receptors and preventing cytokines secretion.

However, some AMPs induce disaggregation on LPS aggregates. This property may favor the antibacterial activity against Gram-negative bacteria and may promote the disruption to Gram-negative bacteria cell wall. For example, Domadia et al. explored the disturbance of LPS aggregates by the interactions with peptide MSI-594 and analogue MSI-594F5A, using dynamic light scattering (DLS). It is found that when LPS was dispersed in the phosphate buffer, the diameter is mainly centered at 7000 nm. However, there is a dramatic shift in the distribution of LPS aggregate sizes in the presence of the peptides [40], which is in accordance with their antimicrobial activity against Gram-negative bacteria. Similarly, the effect of disaggregating LPS aggregates of antimicrobial peptide chensinin-1 is weaker than its analog chensinin-1b, as same as their bacterial activity against Gram-negative bacteria [44].

### 4.3. The Flexible Structures of AMP in LPS and Effect on the LPS Phosphate Groups

Finally, the structure of AMPs also affects their combination with LPS. They exist in different structural forms in LPS environment. It is found that many of the antimicrobial peptides exhibit the random coil structure in aqueous solutions, after interacting with LPS. The secondary structure of antimicrobial peptides changes from random coil to *α*-helix, such as NRC-16 and magainin, and this may be because the amphiphilic structure is more likely to interact with the lipid. Some peptides are in *β*-turn, *β*-chain structure, and *β*-hairpin structure [45]. Bhattacharjya et al. designed a linear peptide YW12 with 12 residues. In aqueous solution, YW12 exists as conformation in the absence of LPS. However, the secondary structure of YW12 transforms from random coil to a well-folded structure in the presence of LPS. N-terminal of YW12 is extended conformation or loop, and C-terminus has two consecutive *β*-turns in LPS. This property makes YW12 easily displaceable in aqueous environment in order to get closer to LPS layer. In addition, the flexible structure is conducive for the interaction with LPS. Tan et al. reported that the S3 peptide goes through conformational changes in the presence of a disulfide bridge, transitioning from a random coil to *β*-sheet structure, with a *β*-sheet conformation binding to the bisphosphorylated glucosamine disaccharide head group of LA, primarily by ion-pair formation between anionic phosphates of LA and the cationic side chains by circular dichroism spectrometry [46]. The *β*-sheet secondary structure of S3 can prolong and continue the interaction with and disruption of LPS micelles [47]. NMR techniques further confirm that cationic C-terminus of melittin uses local coil; hydrophobic N-terminal is unstructured and dynamic in LPS environment. Folded C-terminus acts as the anchor element and disrupts LPS structure. The MSI-594 helix-loop-helix or helix hairpin structure, the compact conformation, can help the AMP translocation across double endotoxin [28]. In conclusion, the random coil structure of AMP is conducive for the movement in aqueous solution, and the well-folded structure in the presence of LPS makes for the further interaction with LPS. One of the target sites is the phosphate groups inside LPS. Raquel Conde and his colleagues found that there are considerable changes in the phosphate as well as the sugar modes of LPS R595 in the presence of PMB. Regarding the phosphates, a drastic decrease of the band intensities at 1257 and 1221 cm^−1^ takes place; the former corresponds to phosphate with low hydration, mainly due to the 4′-phosphate, and the latter band corresponds to phosphate with high hydration, primarily due to the 1-phosphate [48]. The decrease of the intensities can be attributed to a strong reduction of the mobility of both phosphate groups, illustrating that PMB interacts with the phosphate groups of LPS. Since the phosphate groups deeply embed in LPS, the interaction with the phosphate groups is regarded as the index of penetration to LPS barrier, contributing to the effect of antibacterial and anti-inflammation.

## 5. Mechanism of Antimicrobial Peptides Inhibited LPS-Induced Inflammation

LBP plays an important role in LPS-induced inflammation, and it is the trigger for LPS-induced inflammation. The activity of LPS is enhanced by combination with serum LBP. However, AMPs bind to LPS, inhibiting the LPS binding to LBP. Cathelicidins, CAP18 (cationic antibacterial proteins of 18 kDa) and CAP11 (cationic antibacterial polypeptide of 11 kDa), were investigated by Isao Nagaoka et al. These AMPs can bind to LPS and suppress LPS-induced TNF-*α* expression by macrophage cell line RAW264.7. Peptides such as CP29 and Indolicidin block the LPS inflammatory signal transmission by competing with LBP for LPS binding, which reduce, reducing the LPS mediated cytokines TNF-*α* release significantly [49]. When antimicrobial peptides, LPS and LBP, are incubated together, an AMP can successfully prevent LPS combining with LBP but rarely decompose the LPS-LBP complexes. G. Monisha et al. found that, with antimicrobial peptide MBI-28 pretreatment with phagocytic cells for one hour and addition of new culture medium of LPS after removing the supernatant, MBI-28 still suppresses the TNF-*α* expression by macrophages, suggesting that there is another mechanism that inhibits LPS-induced inflammation. MBI-28 may directly interact with immune cell. Yosef Rosenfeld et al. had confirmed that peptide LL37 competes with LPS for immune cell membrane receptor CD14 binding, preventing the binding of LPS and CD14 and inhibiting the release of cytokines [47, 50–52]. This shows that AMP can not only bind to LPS but also interact with immune cell membrane receptor CD14 and therefore inhibit the LPS-induced inflammation. These properties make AMPs the attractive drug candidates for treatment of endotoxin shock and sepsis caused by Gram-negative bacterial infection.

## 6. AMP Function as Innate Immune Regulators

The expression of AMPs is mainly induced by PRR, which can recognize nonspecific, highly conserved PAMPs. LPS of Gram-negative bacteria is one of the most active PAMPs and can promote a resilient induction of the innate immune system. When PRR interacts with PAMP, immune cells secrete chemokines, such as IL-8, monocyte chemotactic protein-1 (MCP-1/3), activating neutrophils, mast cells, and epithelial cells that secrete AMP, like defensins *α* and LL-37 [53, 54]. LL-37 can interact with formyl peptide receptor-like 1 (FPR1) making monocytes, neutrophils, and T lymphocytes chemotaxis. Another research undertaken by Hiemstra et al. showed that after activating FPR1, LL-37 makes leukocyte chemotaxis and enhances the adhesion, phagocytic ability, the release of oxygen, and antibacterial activity, thus strengthening the immune system [55, 56]. AMPs can also induce degranulation of mast cells, prompting the release of histamine and causing vasodilation followed by the release of immune cells in the blood. Consequently, apoptosis of macrophages and activation of lymphocytes were induced. In addition, AMP can enhance the chemotaxis of fibroblasts and proliferation of endothelial cells and lymphocyte and promote wound healing. Niyonsaba et al. found that *β*-defensin-2 cell activation and degranulation of mast cells, followed by the release of histamine and prostaglandin D2, increased permeability of the blood vessel wall [57].

## 7. Conclusion

LPS plays an important role in Gram-negative bacteria-induced inflammation. AMPs not only are intended to kill pathogens through their antimicrobial activity but also have a high affinity for LPS or membrane receptors. Besides, certain AMPs have the essential function of regulating and balancing the inflammatory response of the innate immune system. Though AMPs have the potential to neutralize the endotoxin of LPS to treat infection or inflammation, few of them used for clinical purposes have the stability problem and this needs to be further studied in the future.
